# The effect of bleaching on the optical and physical properties of externally stained monolithic zirconia

**DOI:** 10.1002/cre2.433

**Published:** 2021-06-21

**Authors:** Maryam S. Tavangar, Elaheh Mousavipour, Elham Ansarifard

**Affiliations:** ^1^ Operative Dentistry Department, Dental Faculty Shiraz University of Medical Sciences Shiraz Iran; ^2^ Students' Research Committee, Shiraz University of Medical Sciences Shiraz Iran; ^3^ Department of Prosthodontics, School of Dentistry Shiraz University of Medical Sciences Shiraz Iran; ^4^ Nanobiology and Nanomedicine Research Center Shiraz University of Medical Sciences Shiraz Iran

**Keywords:** color stability, externally stained ceramics, monolithic zirconia, surface hardness, surface roughness, translucency

## Abstract

**Objective:**

This study aimed to investigate the effects of bleaching on the color, translucency, surface roughness, and surface hardness of monolithic zirconia with external stainin .

**Methods:**

In this experimental study, 32 specimens of monolithic zirconia (1 × 1 mm; shade A2) were divided into two groups based on random permuted blocks. Overglaze and staining procedures were performed with a yellow stain or a value stain (GC Stain). Baseline color, translucency, roughness, and surface hardness were measured. The specimens were then randomly bleached with hydrogen peroxide (HP) 40% (20 min, twice with a 1‐week interval in between) as office bleaching or carbamide peroxide (CP) 20% (4 h per day for 14 days) as home bleaching. Finally, the color, translucency, surface roughness, and surface hardness were measured again.

**Results:**

Bleaching with CP and HP caused a perceptible change in the color of the specimens (Δ*E* > 2), although this change was within the clinically acceptable range (Δ*E* < 3.3). HP significantly reduced the surface hardness of the specimens (*p* = 0.043). Changes in surface roughness of the specimens were neither statistically nor clinically significant (*p* = 0.19 and *p* = 0.25 for office and home bleaching, respectively).

**Conclusion:**

The effects of home and office bleaching on the surface characteristics of monolithic zirconia were almost the same. It is not necessary to exchange or even to polish the surfaces of zirconia restorations after exposure to bleaching agents. Further studies are recommended to confirm the color stability of externally stained monolithic zirconia.

## 1 INTRODUCTION

Numerous factors, such as shape, surface texture, position, and color of teeth or dental restorations, affect the appearance of a smile. Ceramic veneers are restorative techniques used to improve the appearance of a smile. Bleaching techniques have also been used to improve the color of teeth (Polydorou, Monting, et al., 2007). In‐office bleaching techniques, commonly performed with high concentrations of hydrogen peroxide (HP), are widely applied because of the benefits such as a rapid response and the protection of soft tissue. Home bleaching techniques are used because of their ease of use, lower concentration of materials, and more sustainable results (Auschill et al., 2005).

Nowadays, in the treatment process, it is quite common for tooth‐colored restorations to be exposed to bleaching agents in the esthetic zone (Polydorou, Monting, et al., 2007). Therefore, many studies have investigated the effect of oxidizing agents within the bleaching materials on the surface and mechanical properties of different tooth‐colored materials. In previous studies, the effects of bleaching agents on a wide range of tooth‐colored restorations including composite resins, glass ionomer, glass modifier resin glass, feldspar porcelain, glass‐reinforced ceramics, and CAD‐CAM manufactured ceramics have been investigated (de Selva et al., 2006; Kara et al., 2013; Karci & Demir, 2019; Polydorou, Monting, et al., 2007; Turker & Biskin, 2003; Vanderlei et al., 2010; Yu et al., 2013).

Conventional dental ceramics, including feldspar porcelain and glass ceramics, present excellent optical and color properties, albeit with low fracture toughness. On the other hand, zirconia‐based ceramics offer excellent mechanical properties. Due to the high opacity of zirconia, veneering with more translucent ceramics is needed. However, chipping of the veneer layer is the most important drawback of these restorations (Shahmiri et al., 2018). With new advances in manufacturing techniques, monolithic restorations can be made as an integrated restoration without the need for veneering. Concurrently with final glazing, the appearance of the restoration can be improved s via external staining to reach a better shade‐matching. However, the most important concern regarding monolithic zirconia is to provide an appropriate color and translucency in conjunction with the adjacent teeth (Kim & Kim, 2014; Shahmiri et al., 2018). Importantly, research indicates that the color stability of the restorations prepared by external staining is questionable over time (Anil & Bolay, 2002; Bativala et al., 1987).

Studies have shown that bleaching agents may compromise the mechanical and surface properties of the restorative materials (Turker & Biskin, 2003; Yu et al., 2013). One important mechanical aspect is surface roughness, which affects the optical properties and color stability of dental materials. Roughened material surfaces increases the free surface energy and enhance bacterial adhesion. Decreasing the surface gloss would result in a dull and non‐esthetic appearance (de Selva et al., 2006). Furthermore, increased roughness would alter the surface hardness, which results in the wear of both the restorative material itself as well as the opposing tooth structures or restorations (de Selva et al., 2006). Surface hardness is another surface characteristic of tooth‐colored restorations that determines the wear resistance or abrasiveness of the restorative materials.

To our knowledge, the effect of bleaching on the optical and surface properties of externally stained monolithic zirconia has not yet been investigated. The purpose of this study was to investigate the effect of two bleaching agents (HP 40% as a common agent used for office bleaching, and carbamide peroxide (CP) 20% as a common agent used for home bleaching) on the color, translucency, hardness, and surface roughness of externally stained monolithic zirconia.

## 2 MATERIALS AND METHODS

### 2.1 Sample preparation

A total of 32 specimens of A2 color zirconia blocks with dimensions of 10 × 10 mm^2^ and a thickness of 1.2 mm were made using a CAD/CAM machine. Sintering was performed using a furnace (LTH 0217, Nabertherm GmbH, Bahnhofstr, Germany) according to factory instructions. The specimens were externally stained using a yellow stain or a value stain (GC Stain) using a synthetic nylon brush according to the manufacturer's recommendations. The colored surfaces were overglazed but were not polished after sintering; they were cleaned with ultrasonication for 5 min before the tests began. After measuring the baseline color, translucency, roughness, and surface micro‐hardness, the specimens of each color were randomly divided into two groups, which were bleached using HP or CP. In overall, the specimens were analyzed within the following four groups (*n* = 8 for each group): (a) value stained zirconia bleached by office bleaching regimen; (b) value stained zirconia bleached by home bleaching regimen; (c) yellow stained zirconia bleached by office bleaching regimen; and (d) yellow stained zirconia bleached by home bleaching regimen. The details of the materials used are presented in Table 1. The specimens were divided into two groups of home and office bleaching.

**TABLE 1:** Materials and agents used in the study

Type		Brand name	Batch number	Manufacturer
Zirconia monolithic	cubic zirconia system 5Y‐TZP Super High Translucent	Dental direct DDCubeX^2^®98	8031848001	Germany
External stain	Value stain	GC Stain	V1332249	USA
	Yellow stain		P128449	
Office bleaching Agent	Opalescence Boost 40% Hydrogen peroxide	ULTRADENT	‐	USA
Home bleaching Agent	Opalescence 20% Carbamide peroxide	ULTRADENT	‐	USA

### 2.2 Bleaching

Specimens of each staining group were randomly divided into two groups and bleached. After 1 day of storage at 37°C and ultrasonic cleaning, all tests were repeated for each sample.

#### 2.2.1 Office bleaching

All specimens were coated with Office Bleaching Gel (40% hydrogen peroxide) with a thickness of 1 mm for 20 min according to the manufacturer's instructions. The specimens were then washed with plenty of water and air‐dried, before being placed in distilled water for 7 days at 37°C. After 7 days, office bleaching was performed again. Then, the specimens were kept in distilled water at 37°C for 24 h ahead of evaluation.

#### 2.2.2 Home bleaching

The specimens were divided and coded into two groups of eight specimens with two different stainings. The specimens were covered with 20% CP for 4 h. Afterward, each sample was washed with high‐pressure water and a soft toothbrush. This process was repeated daily for 14 days. Then, the specimens were kept in distilled water at 37°C for 24 h.

After home and office bleachings were done, the specimens were then washed for 15 min using a 50–60 Hz ultrasonic device with 25–60 Pa water pressure, before finally being air‐dried and all tests were reperformed.

### 2.3 Color and translucency measurements

Color measurement was performed based on the CIE L* a* b* system using the Easyshade V spectrophotometer (VITA, Germany). In this system, L* represents the lightness, a* signifies the inclination of color on the red‐green axis, and b* determines the inclination of color on the blue‐yellow axis. The head diameter of the device was 45 mm and the reflection source included a UV lamp. The specimens were situated in contact with the head of the device during the experiment. The color change (Δ*E*) value was calculated using the following formula with a clinically acceptable range of 1.7 < Δ*E* <3.4 (Ghinea et al., 2010):

$$\Delta E=\left[\Delta a*2+\Delta b*2+\Delta L*2\right]\;½$$

Translucency was evaluated according to the translucency parameter (TP). Measurements were performed three times for each sample and the mean was recorded. The specimens were evaluated using a spectrophotometer once on a white background and again on a black background. The TP was obtained by measuring the color difference of the specimens between the two black and white fields according to the following formula:

$$\Delta \;\mathrm{TP}=\left[\left(L*B-L*W\right)\;2+\left(a*B-a*W\right)\;2+\left(b*B-b*W\right)\;2\right]\;1/2$$

where *B* is black and *W* is white. The higher the TP value, the greater the translucency. The change in TP between before (TP1) after bleaching (TP2) was defined as ΔTP, calculated using the following formula:

ΔTP=TP2−TP1

### 2.4 Surface roughness test

For each sample, surface roughness was measured with a Rugosurf 20 profilometer (TESA, Switzerland) at three different distances and the average (Ra) was reported. The head of the machine was calibrated to 0.75 μm.

### 2.5 Surface hardness test

Using the Vickers hardness test, microhardness was measured at a pressure of 2.94 Newton and a dwell time of 30 s at 3 points on each sample with the MHV‐1000Z (SCTMC Company, China) device. The two diagonals of the pyramid (*d*_1_ and *d*_2_) created by the indenter were measured. Then the amount of Vickers hardness for each specimen was calculated by the follwing formula:

$$\mathrm{HV}=1.854\times F/d^{2}$$

In which the *F* represents load in Load in kgf and *d* is the arithmetic mean of the two diagonals, *d*_1_ and *d*_2_ in mm. The average hardness obtained from the three zones was recorded as sample microhardness.

### 2.6 Statistical analysis

Taking into account power of 80%, a level of confidence of 95%, an effect size of 50% (before and after Polydorou, Monting, et al., 2007), and a correlation of 0.5, the sample size was determined to be 32. The data obtained from the tests performed on the specimens were entered into SPSS software version 19 (IBM). The normality of distribution was analyzed. To test for significant differences between groups with normal distribution, analysis of variance (ANOVA; for multiple groups) and the independent sample *t*‐test (for two groups) were used. The Kruskal–Wallis (for multiple groups) and Mann Whitney (for two groups) tests were used for comparison between groups when data were not normally distributed. The paired sample *t*‐test was used for comparison of before/after changes. Results with *p*‐values less than 0.05 were considered statistically significant.

## 3 RESULTS

### 3.1 Color and translucency change

Evaluation of office and home bleaching for both the yellow and value stain groups of monolithic zirconia revealed changes in color within the detectable range (Δ*E* ≥1.7). All values of Δ*E* were greater than two but remained within the clinically acceptable range (1.7 < Δ*E* <3.4).

The comparison of color changes between the four groups revealed that the numerical values of color change were higher in the yellow stain groups than the value stain groups, but without any significant difference (*p* = 0.554). Also, the comparison of changes in translucency and color components (a*, b*, and L*) between the four groups revealed no significant difference. However, the numerical values of translucency decreased in the value stain groups but increased in the yellow stain groups (Table 2).

**TABLE 2:** Comparison of color and translucency changes

	Δ*E*	Δ*a*	Δ*b*	Δ*L*	ΔTp
Office value	2.06 ± 0.402	0.30 ± 0.33	−0.69 ± 0.28	−1.062 ± 0.58	−0.724 ± 0.74
Office yellow	2.71 ± 0.568	−0.41 ± 0.27	−0.42 ± 0.46	−0.762 ± 0.97	0.782 ± 0.39
Home value	2.28 ± 0.378	0.00 ± 0.17	0.77 ± 0.26	1.675 ± 0.55	−1.019 ± 0.98
Home yellow	2.81 ± 0.398	0.48 ± 0.22	0.14 ± 0.60	−1.0 ± 0.84	0.112 ± 1.15
*p*‐Value	0.554	0.067	0.052	0.069	0.583

Comparison the values of a*, b*, and L* before and after the intervention within each of the four groups showed some significant values of b* and L* (Table 3; *p* = 0.022 and *p* = 0.018 respectively).

**TABLE 3:** Comparison of color changes before and after intervention

	Group	Before	After	*p*‐Value
L*	Office value	72.95 ± 1.19	71.91 ± 1.22	0.118
	Office yellow	59.15 ± 1.30	58.38 ± 0.84	0.459
	Home value	68.17 ± 1.05	69.86 ± 1.04	0.018*
	Home yellow	57.41 ± 1.71	56.41 ± 1.75	0.273
*a* *	Office value	1.56 ± 0.38	1.86 ± 0.38	0.390
	Office yellow	5.77 ± 0.67	5.36 ± 0.69	0.165
	Home value	1.60 ± 0.34	1.57 ± 0.29	0.876
	Home yellow	6.08 ± 0.48	6.56 ± 0.36	0.068
*b* *	Office value	27.40 ± 2.56	26.71 ± 2.47	0.043^*^
	Office yellow	43.51 ± 1.47	43.08 ± 1.54	0.390
	Home value	24.40 ± 2.22	25.16 ± 2.39	0.022^*^
	Home yellow	44.43 ± 1.64	44.58 ± 1.54	0.812

*Note*: Data are represented as mean ± SE.

^*^ *p*‐value <0.05.

Then, to compare the two bleaching methods irrespective of staining, an analysis was performed between the office and home groups. There were no significant differences between the two bleaching methods in terms of color and translucency changes. Notably, the values of the color components (a*, b*, and L*) decreased with office bleaching and increased with home bleaching, though only Δb* showed a significant difference between the two groups (*p* = 0.022; Table 4).

**TABLE 4:** Comparison of color and translucency changes

Office average	2.38 ± 0.34	−0.056 ± 0.15	−0.556 ± 0.26	−0.912 ± 0.55	0.029 ± 0.45
Home average	2.55 ± 0.27	0.260 ± 0.22	0.456 ± 0.32	0.337 ± 0.59	−0.453 ± 0.75
*p*‐Value	0.712	0.255	0.022^*^	0.134	0.583

*Note*: Data are represented as mean ± SE. Δ*E*, color change; ΔTp, transparency change.

^*^ *p*‐Value <0.05.

### 3.2 Roughness

**FIGURE 1:**
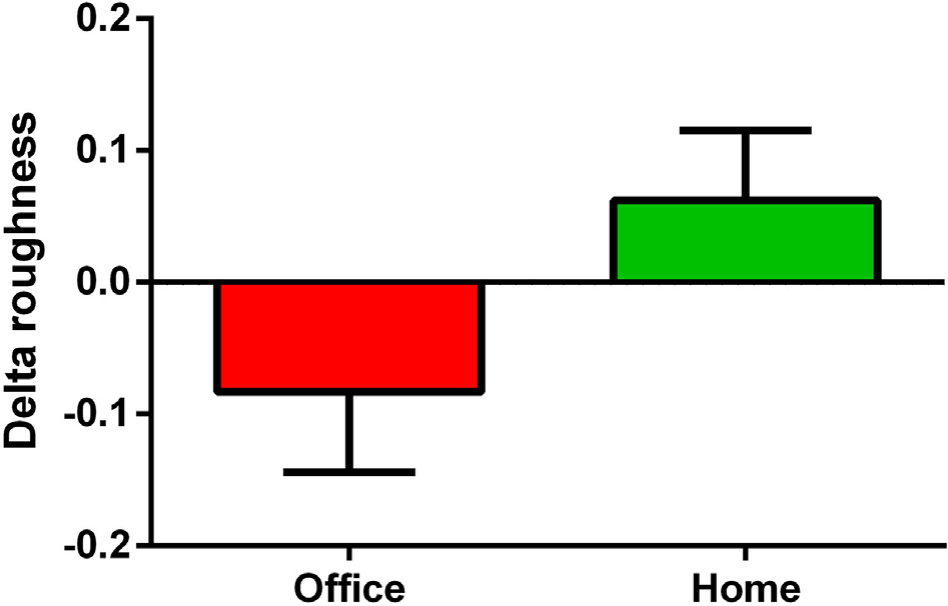
Comparison of roughness change between office and home bleaching (*p* = 0.084)

There was a non‐significant decrease in roughness with office bleaching (*p* = 0.197) but an increase in roughness with home bleaching (*p* = 0.258, Table 5). The comparison of roughness changes between the two groups of office and home bleaching revealed no significant difference (*p* = 0.084, Figure 1). The changes in surface roughness did not correlate with changes in color and translucency (Table 6).

**TABLE 5:** Comparison of roughness and hardness before and after intervention

	Group	Before	After	Change	*p*‐Value
Surface roughness (μm)	Office	0.37 ± 0.06	0.287 ± 0.03	−0.083 ± 0.06	0.197
	Home	0.284 ± 0.04	0.347 ± 0.03	0.063 ± 0.05	0.258
Micro‐hardness (kg/mm^2^)	Office	524.25 ± 19.42	474.08 ± 27.34	−50.171 ± 22.69	0.043^*^
	Home	494.76 ± 20.96	510.1 ± 17.03	15.343 ± 10.08	0.149

*Note*: Data are represented as mean ± SE.

^*^ *p*‐Value <0.05.

**TABLE 6:** Correlations between different variables

			Roughness	Δ*E*
Office	**Δ** *E*	*r*	−0.294	
		*p* Value	0.270	
	**Δ**Tp	*r*	−0.223	0.203
		*p* Value	0.406	0.450
Home	**Δ** *E*	*r*	−0.165	
		*p* Value	0.542	
	**Δ**Tp	*r*	−0.035	0.233
		*p* Value	0.899	0.385

### 3.3 Hardness

**FIGURE 2:**
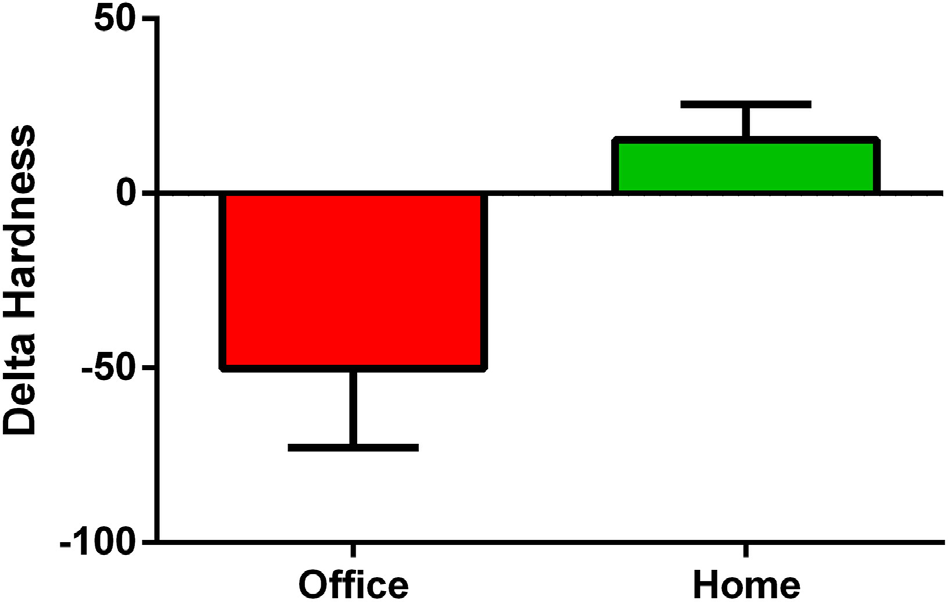
Comparison of hardness change between office and home bleaching. The difference was significant (*p* = 0.013)

The statistical analysis showed a significant decrease in hardness with office bleaching (*p* = 0.043) and a non‐significant increase in hardness with home bleaching (*p* = 0.149, Table 5). The comparison of hardness changes between the two groups of office and home bleaching showed a significant difference (*p* = 0.013, Figure 2).

## 4 DISCUSSION

In the current study, the authors investigated the effect of bleaching on the color, translucency, hardness, and surface roughness of externally stained zirconia. Here, the authors found that bleaching externally stained monolithic zirconia using CP or HP resulted in color change (Δ*E*) values of greater than two, which were clinically acceptable (<3.4) but within the perceptible range (1.7 < Δ*E* < 3.4) of color change (Ghinea et al., 2010).

Here, we did not find a significant color change difference between HP and CP bleaching. The comparison of the color changes between staining groups also yielded no results of significance, although the numerical values were higher in the yellow stain group compared to the value stain group. In a study by Kara et al., the effects of 10% HP and 10% CP on the lithium‐disilicate‐based all‐ceramic (IPS Empress 2 and IPS Empress e‐Max), ultralow‐fusing porcelain (Finesse), and low‐fusing porcelain (VITA VM 9) specimens were investigated. They found that the most noticeable color change was obtained in the IPS Empress II group (Δ*E* = 1.60 after bleaching with HP and 1.66 after bleaching with CP). In the comparison of the effect of HP and CP, they showed a significant difference in the two groups of ceramics (Kara et al., 2013). The degree of color change in our study was greater than those noticed in the Kara et al. study, which can be attributed to the higher concentration of bleaching agents used in our study (HP 40% and CP 20%). Another explanation of the observed differences may be related to the point that restorations were externally stained in our study, which has been shown in other studies to affect color stability. In one study, Anil et al. investigated the effect of tooth brushing on the color stability of intrinsically and extrinsically stained porcelain. They suggested that staining should be done as deeply as possible to achieve a durable color (Anil & Bolay, 2002). In another study, Kanat‐Erturk et al compared the color stability of two types of CAD/CAM ceramics (lithium disilicate and zirconia‐reinforced lithium disilicate) prepared with three different surface finishing methods (glazing, mechanical polishing, and external staining accompanied by glazing) after storage in black tea and coffee. They noticed that lithium disilicate ceramics finished by external staining and glazing depicted lower color stability compared with glazing alone, whereas zirconia‐reinforced lithium disilicate ceramics showed no significant difference in color between external staining and glazing alone (Kanat‐Ertürk, 2020).

The whitening effect of bleaching agents on dental materials may be accompanied by increased opacity. In a study on types of leucite‐reinforced glass–ceramic and lithium disilicate glass–ceramic, it was reported that CP 16% bleaching significantly reduced the translucency of these all‐ceramic restorations (Karci & Demir, 2019). In our study, we noticed no significant difference in translucency before and after bleaching and also between the different bleaching methods.

In our study, there was a trend toward a reduction of roughness with HP 40% (−0.083 μm) but an increase with CP 20% (+0.063 μm). These roughness changes were not statistically significant and were less than the clinically important cutoff point of 0.30 μm (de Selva et al., 2006). In several studies, it has been shown that CP gel at different concentrations, including 10, 16, and 35%, increased the surface roughness of dental ceramics (Moraes et al., 2006; Rea et al., 2019; Vanderlei et al., 2010). However, in Turker et al's study, no changes were observed with CP 10% gel at three different pH values (Turker & Biskin, 2003).

In several studies using HP (38 or 40%) or CP (10, 15, or 16%), no significant change was observed in the post‐bleaching surface microhardness of ceramics (Ourique et al., 2011; Polydorou et al., 2006; Polydorou, Hellwig, & Auschill, 2007; Polydorou, Monting, et al., 2007; Yu et al., 2011; Yu et al., 2013). There was also no evidence of dissolution and loss of matter from the surface of the material (Polydorou, Monting, et al., 2007). In our study, there was a significant reduction of surface micro‐hardness with HP 40% (*p* = 0.043) but an increasing trend in hardness with 20% CP bleaching. Also, the comparison of the two bleaching techniques showed a significant difference in terms of their effect on surface micro‐hardness. Contrary to our findings, Malkondu et al. found that CP significantly reduced the surface microhardness of three ceramics including leucite‐reinforced glass–ceramic, glass–ceramic, and feldspathic porcelain, while HP 10% exerted no significant effect (Malkondu et al., 2011). In a study by Turker and his colleagues on feldspar porcelains, a decrease in microhardness after bleaching with CP was shown (Turker & Biskin, 2003). In another study by the same researchers, the findings showed a 4.8 and 4.4% decrease in SiO2 content in the presence of 16 and 10% CP, respectively (Turker & Biskin, 2002). These controversies may go back to the differences in the concentrations of the bleaching agents, ceramic type, and type of ceramic surface conditions. Passos and colleagues have shown that the concentration of bleaching agents and the type of ceramics significantly influence the changes in microhardness (Passos et al., 2010). Torabi et al. depicted that the hardness of overglazed or polished surfaces is affected more by exposure to bleaching agents compared to autoglazed ceramic surfaces (Torabi et al., 2014).

Some studies have evaluated the susceptibility of ceramics to the colorant effects of beverages like coffee and tea (Culic et al., 2017; Kanat‐Ertürk, 2020). A limitation of our study was that we did not store our ceramics before or after bleaching in such colorant media. Consequently, our study fails to address the issue that whether or not monolithic zirconia becomes more susceptible to staining after bleaching; this remains to be investigated in the future.

## 5 CONCLUSION

In conclusion, bleaching with CP 20% or HP 40% could perceptibly change the color of externally stained monolithic zirconia, albeit within a clinically acceptable range. Furthermore, HP significantly decreased the surface micro‐hardness, though no significant clinical or statistical change was noted in the surface roughness of the externally stained monolithic zirconia after bleaching.

## CONFLICT OF INTEREST

The authors declare they have no conflict of interest.

## Data Availability

Data will be available by the corresponding author under reasonable request.
